# Emotional disorders evidenced by family caregivers of older people with Alzheimer’s disease

**DOI:** 10.1590/1980-57642020dn14-010009

**Published:** 2020

**Authors:** Carlene Souza Silva Manzini, Francisco Assis Carvalho do Vale

**Affiliations:** 1 Universidade Federal de São Carlos Ringgold standard institution – Enfermagem – Avenida Rio de Janeiro, Araraquara, SP Brazil.; 2 Universidade Federal de São Carlos Ringgold standard institution – Medicina Sao Carlos – São Paulo, SP, Brazil.

**Keywords:** Alzheimer’s disease, aged, caregivers, family, dementia, geriatric nursing, doença de Alzheimer, idoso, cuidadores, família, demência, enfermagem geriátrica

## Abstract

The task of caring for a family member with dementia is associated with caregiver physical and emotional problems. The patient’s decline in health and specific needs contribute directly to this situation.

**Objective:**

To evaluate burden, stress, depression and anxiety symptoms in family caregivers of elderly with Alzheimer’s disease.

**Methods:**

A cross-sectional, descriptive, correlational and quantitative study was carried out. The sample consisted of 66 family caregivers of elderly with Alzheimer’s disease, whom attended the Cognitive and Behavioral Neurology Outpatient Clinic of the Federal University of São Carlos, in the city of São Carlos, SP, Brazil.

**Results:**

Of the caregivers evaluated in the severe AD subgroup, 47.3% had intense burden; 86, 4% exhibited significant stress levels; 57% presented severe anxiety levels and 36.9% presented mild depression symptoms.

**Conclusion:**

Caring for a family member with Alzheimer’s disease generates burden, stress, anxiety and depression. Support groups comprising a multiprofessional team can be set up to assist caregivers. These actions can help caregivers cope with the daily demands and challenges and ensure better care quality in an increasingly aging population.

## RESUMO

A tarefa de cuidar de um familiar com demência está relacionada a problemas físicos e emocionais no cuidador. O declínio do paciente e suas demandas específicas contribuem diretamente para essa situação.

**Objetivo:**

Avaliar sintomas de sobrecarga, estresse, depressão e ansiedade em cuidadores familiares de idosos com doença de Alzheimer.

**Métodos:**

Estudo transversal, descritivo, correlacional e de caráter quantitativo. A amostra foi composta por 66 cuidadores familiares de idosos com doença de Alzheimer, atendidos no Ambulatório de Neurologia da Universidade Federal de São Carlos, na cidade de São Carlos, SP, Brasil.

**Resultados:**

Dos cuidadores alocados no subgrupo DA avançada, 47,3% foram avaliados com sobrecarga intensa; 86,4% apresentaram níveis de estresse significativo; 57% apresentaram níveis graves de ansiedade e 36,9% apresentaram sintomas de depressão leve.

**Conclusão:**

Cuidar de um familiar com doença crônica e degenerativa como a doença de Alzheimer gera sobrecarga, assim como estresse, ansiedade e depressão. Grupos de apoio compostos por uma equipe multiprofissional podem ser implantados no sentido de garantir o acompanhamento desses cuidadores. Tais ações podem auxiliar a lidar com as demandas e os enfrentamentos diários e ainda garantir uma melhor qualidade no cuidado prestado, considerando o avanço do envelhecimento populacional.

Dementia is a syndrome characterized by cognitive or behavioral symptoms (neuropsychiatric symptoms) that interfere with work or other activities of daily living and represent a decline in previous level of functioning, which is not explained by delirium or severe psychiatric disorders.^1^

The most common type of dementia is Alzheimer’s Disease (AD), which poses significant epidemiological, economic and social impacts.^2^ AD is a progressive and degenerative disease of the brain affecting numerous cognitive areas resulting in declines in functional ability and behaviour changes.^3^ AD compromises the physical, mental and social integrity of the elderly, triggering a dependence situation that requires care, most often integral and complex, performed invariably by a family member.

Caring for a family member with dementia is a task recognized as being associated with caregiver physical and emotional problems. The patient’s decline and specific needs contribute directly to this situation. In a meta-analysis of 33 studies about gender differences in caregivers, the authors demonstrated that female caregivers reported having more care hours, high levels of burden and depression, and lower levels of physical health and subjective well-being than male caregivers.^4^

Caring for another involves a sense of stress related to unpaid and informal work, thus done at home, often without any recognition, even by the family. Regarding family caregivers, they must be able to recognize their limits both for their intervention and for the patient’s, as prolonged involvement in informal care may have a negative effect on the physical and emotional health of the carer.^5^

The health team that assists the elderly with AD needs to expand their assistance to family caregivers and develop a care plan to favor life quality improvement and prevent emotional disorders arising from the care process. Thus, investigations are still needed to measure the emotional disorders evidenced by family caregivers, within clinical practice and research.

This study aimed to evaluate the symptoms of psychological burden, stress, depression and anxiety in family caregivers of elderly with AD. The findings can contribute to knowledge in this area and suggest new directions for devising public policies to promote caregiver health and prevent diseases in this population.

## METHODS

This is a cross-sectional, descriptive, correlational and quantitative study. The research was conducted at the Cognitive and Behavioral Neurology Outpatient Clinic of the Federal University of São Carlos, in the city of São Carlos, SP, Brazil. The Cognitive and Behavioral Neurology Outpatient Clinic is a specialized neurological medical outpatient clinic that assists people with cognitive and behavioral disorders associated with neurological and non-neurological diseases, especially dementias. The data were collected from May to October of 2014. The study included family caregivers who lived with and cared daily for the elderly, who had been caring for more than six months, involving patients over 18 years of age, male or female, with any level of education. The exclusion criterion of the research was refusal by the participant to sign the Free and Informed Consent Form.

The sample size was 66 subjects, distributed according to disease stage. The dementia phases were verified using the Clinical Dementia Rating Scale (CDR). The AD Group comprised 66 family caregivers divided into three subgroups according to the stage of dementia:

a) Mild AD subgroup – 25 caregivers, the elderly in mild phase dementia.
b) Moderate AD subgroup – 22 caregivers, the elderly in moderate phase dementia.
c) Severe AD subgroup – 19 caregivers, the elderly in severe phase dementia.

All the participants signed the Informed Consent Form. This project was approved by the Research Ethics Committee of the Federal University of São Carlos, under permit no. 489,795 and complied with resolution 466/12 for research involving humans.

For data collection, the following instruments were used: a caregiver characterization questionnaire: prepared by the researchers, which collected personal information, lifestyle and care characteristics; the Zarit Burden Scale-ZBS,^6^ this evaluates the main caregiver burden on different levels. The higher the score, the higher the burden perceived by the caregiver. The scale is scored as follows: intense burden scores, 61-88; moderate to severe, 41-60, moderate to mild, 21-40; and absence of burden scores < 21 points; Self Report Questionnaire - SRQ-20,^7^ whose objective is to evaluate emotional discomfort and stress in the general population. The questions in the scale have two possible responses (Yes = 1 point, and No = 2 points) and are designed to screen emotional and physical symptoms. The score ranges from 0 to 20 ( ≤ 7 = non-significant emotional stress; ≥ 8 = significant emotional stress); the Beck Anxiety Inventory-BAI,^8^ a self-administered questionnaire used to assess characteristic anxiety symptoms. The instrument has a score ranging from 0 to 63 points, where 0-7 indicates minimum anxiety; 8-15 mild anxiety; 16-25, moderate anxiety; and 26-63 indicates severe anxiety; the Beck Depression Inventory-BDI,^9^ is an instrument used to screen depressive symptoms. The final score can be classified as: ≤ 9 points, indicating the individual is not depressed, 10-18 points indicates mild depression, 19-29 points moderate depression and 30-63 points indicates severe depression. All instruments were adapted and validated to Brazilian culture.

Data were processed through descriptive statistics and expressed in a frequency table, with absolute values (n) and percentages (%) for categorical variables, and with position and dispersion measures for continuous variables. The Shapiro-Wilks test was used to verify the normality of the data, and the Kruskal-Wallis test was used to estimate the differences among the three groups. The significance level adopted was 5%.

## RESULTS

Table 1 presents the sociodemographic data from the 66 family caregivers that comprised the sample. The caregiver group was analyzed as a whole (all AD) or by taking subgroups individually classified according to dementia stage (mild AD, moderate AD, severe AD). In the all AD group, most caregivers were female, married or had a stable union, a mean age of 56.0 (± 13.3) years and mean education level of 8.0 years. Further sociodemographic data are given in Table 1.

**Table 1:** Demographic characteristics of the family caregivers of elderly with AD (n=66). São Carlos, SP, Brazil. 2015.

Variable	Mean	SD	Distribution of categories	n	%
Sex	--	--	Female	40	60.6
			Male	26	39.3
Age	56.0	(± 13.3)			
Marital status	--	--	Married	50	75.7
			Divorced	11	16.6
			Widowed	3	4.5
			Single	2	1
Education (years)	8.06	(± 3.9)			
Level of kinship	--	--	Daughter/Son	34	51.5
			Spouse	20	30.3
			Sister/Brother	3	4.5
			Nephew/Niece	3	4.5
			Daughter/Son-in-law	3	4.5

SD: standard deviation.

Regarding the time spent on care, caregivers in the severe AD subgroup had highest average hours/day of care (11.5 h), caregivers in the moderate AD subgroup cared for an average of 9.8 hours/day, and the mild AD subgroup provided an average of 7.9 hours/day of care.

The more advanced the dementia stage, the greater the caregiver burden. The most burdened caregivers were those in the severe AD subgroup, 47.3% of whom were evaluated as having severe burden, as shown in Table 2.

**Table 2:** Distribution of burden levels of family caregivers according to ZBS.^6^ São Carlos, SP, Brazil. 2015.

	Severe			Moderate			Mild			Absent	
	N	%		n	%		n	%		n	%
Mild AD	4	16.0		6	24.0		11	44.0		4	16.0
Moderate AD	6	27.2		10	45.4		5	22.8		1	4.6
Severe AD	9	47.3		4	21.1		3	15.8		3	15.8
All AD	19	28.8		20	30.3		19	28.8		8	12.2

In the all AD group, and in its subgroups, there were more caregivers whose stress levels were rated as significant than non-significant. The highest prevalence was found in the severe AD group (94.8%), as seen in Table 3.

**Table 3:** Distribution of stress levels of family caregivers according to SRQ.^7^ São Carlos-SP, Brazil. 2015.

	Non-significant			Significant	
	n	%		n	%
Mild AD	6	24.0		19	76.0
Moderate AD	2	9.0		20	91.0
Severe AD	1	5.2		18	94.8
All AD	9	13.6		57	86.4

Based on Table 4, most caregivers, except the subgroup of mild AD caregivers, had a severe anxiety level. The rate of this anxiety level was highest among moderate AD caregivers (63.6%), followed by severe AD caregivers.

**Table 4:** Distribution of anxiety levels of family caregivers according to BAI^8^ São Carlos-SP, 2015.

	Severe			Moderate			Mild	
	n	%		n	%		n	%
Mild AD	12	48.0		13	52.0		0	0.0
Moderate AD	14	63.6		8	36.4		0	0.0
Severe AD	12	63.2		6	31.5		1	5.3
All AD	38	57.5		27	41.0		1	1.5

Of the all AD caregiver group, 31.9% had symptoms suggestive of mild depression and 6.0% moderate depression, according to Beck’s Depression Inventory. In the severe AD caregiver subgroup, 36.9% presented symptoms suggestive of mild depression, followed by the moderate AD subgroup with 36.4% mild depression and the mild AD subgroup with 24.0% (see Table 5).

**Table 5:** Distribution of depression levels of family caregivers according to BDI.^9^ São Carlos-SP, Brazil. 2015.

	Moderate			Mild			Absence	
	n	%		n	%		n	%
Mild AD	2	8.0		6	24.0		17	68.0
Moderate AD	1	4.5		8	36.4		13	59.1
Severe AD	1	5.2		7	36.9		11	57.9
All AD	4	6.0		21	31.9		41	62.1

## DISCUSSION

In the present study, most caregivers were female and had a mean age of 56.0 years, consistent with data observed in other studies.^5^^,^ 10 ^,^ 11 Caregivers’ average education level was 8.0 years of schooling, results evidenced in national studies.^12^^,^ 13 However, in an international study carried out in Faro (Portugal) involving 110 informal caregivers for dependent elderly found that most caregivers (67.3%) had four years or less of education, characterizing a low education profile.^14^ People with a lower educational level may be susceptible to the caregiver role, as society requires higher educational levels in the formal labor market. Thus, it is understandable that family members with less formal education dedicate time to housework and caring for their dependent family members.

Regarding marital status, the present study indicated that most caregivers were married and consisted of mostly daughters and wives. The results of the present study are also consistent with a study conducted in elderly with AD caregivers at the University of São Paulo Medical School, showing that 64% of caregivers were married.^15^ Having a stable union can translate to benefits for caregivers, since they may recruit their partner´s support for activities with the dependent elderly and are less prone to feelings of loneliness, contributing to better physical and subjective well-being.

Regarding the time spent on care, caregivers of the severe AD subgroup had higher average hours/day of care (11.5 h). The time spent on care increases with worsening dementia. The task of caring for a family member with AD requires almost exclusive dedication, as evidenced in this research by the time caregivers practiced daily care. Often caregivers do not have time to take care of themselves, leaving aside their other tasks to provide care, results corroborated by another study in which caregivers reported giving up time in their own life to care for the sick.^16^

According to scores on the ZBS,^6^ the all AD group had moderate or intense burden. The most burdened caregivers were from the severe AD subgroup, in which 47.3% were evaluated as having severe burden. A prospective observational study conducted in France, Germany and the United Kingdom described the characteristics of 1497 informal community caregivers of patients with severe AD. The results showed median mild (n = 597) to moderate (n = 472) burden.^17^ When the caregiver is able to recognize AD changes and its stages, many problems can be avoided and burden can be minimized. Moreover, the presence and severity of behavioral symptoms in the elderly with dementia are associated with a higher level of caregiver burden.^18^^,^ 19

According to a recent meta-analysis, those caring for people with dementia experience greater burden. Furthermore, subjective caregiver burden is a significant risk factor for depressive symptoms in carers of older people who may develop clinical depression. The authors suggest the use of interventions aimed at alleviating subjective caregiver burden to prevent depressive symptoms and psychiatric morbidity in this population, where cognitive reappraisals, teaching of coping strategies and provision of emotional support, are effective for reducing caregiver burden and may protect carers’ mental health by reinforcing protective psychological mechanisms.^20^

In the present study, caregivers were significantly stressed, as exemplified in the severe AD subgroup case, presenting stress in 94.8% of caregivers. These results prove the emotional burden that the disease causes family members, especially family caregivers. AD is a problem that especially affects the caregivers’ personal and family life. They may feel depressed, anxious, and stressed because of the care they provide, often without support from other family members or even other support types. The literature shows that the role of caring is directly associated with stress, since it impacts the health, family balance and quality of life of those who perform it.^21^

Practicing and maintaining care for a family member with a chronic disease, especially a progressive and neurodegenerative condition such as AD, requires the good physical and mental health of the caregiver, since the patient´s needs call for an increasing level of attention and care as the disease stage progresses. Routine attrition may occur due to lack of support to caregivers, lack of knowledge held by caregivers about dementia stages and lack of preparedness for care.^22^

Regarding anxiety level, there was a high score among caregivers; the rate of severe anxiety was highest in the moderate AD subgroup, followed by the severe AD subgroup. In this respect, the literature shows that caregivers may present a high anxiety level, caused either by care activity burden or by their family structure caused through social roles shifting.^13^ Other aspects may be involved in the caregiver’s feeling of anxiety, such as lack of knowledge about dementia, its severity, fear of loss, daily and uninterrupted involvement associated with the provision of care.

The present study showed that less than half of the caregivers in the all AD group had symptoms suggesting mild and moderate depression. Study participants were family caregivers who lived with and cared for the elderly on a daily basis and had been caring for over six months. Providing informal care can lead to mental health problems such as stress, fear, depression, and concerns about the future and the caregiving tasks.^23^

Studies have demonstrated that, over time, as the elders dependence on care increases, the difficulties of taking care of another person may create burden and feelings of stress, and may contribute to caregiver depression, affecting their quality of life.^24^^,^ 25

The study was limited by the small sample size, precluding the generalization of results to the general population. For future research, comparisons between experimental and control groups are suggested.

In conclusion, the study results showed the occurrence of burden, stress, anxiety and depression among family caregivers of elderly with AD. In general, the literature summarizes these disorders in physical and financial burden, which tends to worsen with disease evolution, lack of information, little social support resources, and few sources of emotional support.

Support groups comprising multiprofessional teams can be set up to assist caregivers. These actions can help carers cope with daily demands and challenges, taking preventative actions, thereby ensuring better care quality in an increasingly aging population.
